# The Association of Dietary Fiber Intake in Three Meals with All-Cause and Disease-Specific Mortality among Adults: The U.S. National Health and Nutrition Examination Survey, 2003–2014

**DOI:** 10.3390/nu14122521

**Published:** 2022-06-17

**Authors:** Jiayue Qi, Jian Gao, Yuntao Zhang, Wanying Hou, Tianshu Han, Changhao Sun

**Affiliations:** National Key Discipline, Department of Nutrition and Food Hygiene, School of Public Health, Harbin Medical University, 157 Baojian Road, Harbin 150081, China; 202001043@hrbmu.edu.cn (J.Q.); 201901043@hrbmu.edu.cn (J.G.); 202001041@hrbmu.edu.cn (Y.Z.); 2020020239@hrbmu.edu.cn (W.H.); 2019020117@hrbmu.edu.cn (T.H.)

**Keywords:** chrono-nutrition, dietary fiber, mortality, NHANES

## Abstract

The timing of food intake can significantly alter the body’s metabolism of nutrient intake and affect the occurrence of chronic diseases. However, whether and how the intake time of dietary fiber could influence mortality risks is largely unknown. This study aims to reveal the association between total dietary fiber intake and fiber intake at different times with all-cause, cancer, and cardiovascular disease (CVD) mortality rates. A total of 31,164 adults who enrolled in the National Health and Nutrition Examination Survey from 2003 to 2014 are included in this study. Dietary fiber intake was measured using 2-day, 24 h dietary recall. The main exposures in this study were the intake of dietary fiber at breakfast, lunch, and dinner via regression analysis of the residual method. The main outcomes were the all-cause, cancer, and CVD mortality rates. Cox proportional hazards regression models were used to evaluate the survival relationship between dietary fiber intake at different times and mortality rates. Among the 31,164 adults, 2915 deaths, including 631 deaths due to cancer and 836 deaths due to CVD, were documented. Firstly, after adjusting for potential confounders, compared to the participants in the lowest quintile of total dietary fiber intake, the participants in the highest quintile of fiber intake had lower all-cause (HR = 0.686, 95% CI: 0.589–0.799, *p* for trend <0.001) and cancer (HR = 0.606, 95% CI: 0.446–0.824, *p* for trend = 0.015) mortality risks. Secondly, compared to the participants in the lowest quintile of dietary fiber intake at dinner, the participants in the highest quintile of fiber intake had lower all-cause (HR = 0.796, 95% CI: 0.668–0.949, *p* for trend = 0.009) and cancer (HR = 0.564, 95% CI: 0.388–0.822, *p* for trend = 0.005) mortality risks. Furthermore, equivalently replacing each standard deviation of dietary fiber consumed at breakfast with that at dinner was associated with lower cancer mortality risks (HR = 0.846, 95% CI: 0.747–0.958). In conclusion, this study demonstrates that, in the NHANES (2003–2014) cohort, to reduce all-cause and cancer mortality risks, the optimal dietary fiber intake time is in the evening.

## 1. Introduction

Human dietary activity is a complex behavior. In addition to the quantity and quality of food intake, current studies have found that the timing of food intake can significantly alter the body’s metabolism of nutrient intake and affect the occurrence of chronic diseases [1]. As a result, research in the field of nutrition has created the concept of chrono-nutrition, which posits that when you eat is as important as what you eat [2]. A growing body of animal and human studies have shown that the timing of energy, macronutrient, and micronutrient intake had an influence on biological clocks and circadian rhythms, which are associated with the development of cancer and CVD [3,4,5,6,7]. However, to date, whether and how the intake time of dietary fiber could impact the health of humans remains largely unknown. Generally speaking, dietary fiber is made up of edible plant parts or similar carbohydrates that resist digestion and absorption in the small intestine and can be fermented by the large intestine microbiota, producing short fatty acids that have the beneficial physiological effects of promoting bowel movements and lowering blood glucose and blood cholesterol levels [8,9]. Several cohort studies have shown that high daily dietary fiber intake reduced the risk of all-cause, CVD, and cancer mortality [10]. A meta-analysis of observational studies suggested that dietary fiber intake is inversely associated with the risk of esophageal cancer [11]. A random-effects meta-analysis of prospective observational studies demonstrated that high total fiber consumption was associated with a reduced risk of breast cancer [12]. A pooled analysis of prospective cohort studies indicated that the association between dietary fiber intake and risk of colorectal cancer is inverse [13]. In addition, numerous epidemiological studies have shown that increased dietary fiber intake was significantly associated with reduced risks of obesity, CVD, and diabetes [14,15,16,17,18,19,20], which are major causes of death and risk factors for several cancers. Furthermore, a series of animal studies have found that dietary fiber metabolites produced via intestinal fermentation were associated with cancer-related homeostasis mechanisms, including inflammatory response, oxidative stress, immune response, and cancer cell apoptosis [21,22,23]. Recent studies showed that intestinal microbiota present diurnal oscillations that are synchronized with the host’s circadian clock and feeding rhythms [24]. Gut microbiota ferments dietary fibers and produces short-chain fatty acids as possible zeitgebers or synchronizers of peripheral circadian clocks, which could modulate the expression of clock genes in peripheral tissues and food-induced peripheral clock entrainment in mammals [25], and it could cause the host circadian clock network to affect the host physiology metabolism [26].

Based on the above-mentioned evidence, we hypothesized that a correct and regular dietary fiber intake time was necessary to regulate the body’s metabolism and affect health. This study analyzes the association of total dietary fiber intake and fiber intake at different times with all-cause, cancer, and CVD mortality rates using data from the National Health and Nutrition Examination Survey (NHANES) 2003–2014.

## 2. Methods

### 2.1. Study Population

The NHANES is a stratified, multistage study that uses a nationally representative sample of the population of the U.S.; detailed information regarding this study has been described elsewhere [27]. For all of the participants, a home interview was followed by an examination in a mobile examination center to collect various health- and nutrition-related data. The study gained institutional ethics review board approval from the National Center for Health Statistics, and written informed consent was obtained from each participant before data collection. The survey data and questionnaires are available to be downloaded from the National Health and Nutrition Examination Survey website: http://www.cdc.gov/nchs/nhanes.htm (accessed on 27 May 2022). Briefly, participants who finished at least 1 valid dietary recall of the NHANES from 2003 to 2014 were selected to be used in this study, and those who were <18 years, had total energy intake >4500 kcal/d or <500 kcal/d, and had missing information regarding any dietary intake, BMI, and/or mortality aspects were excluded from the study. A total of 31,164 participants were included in this study, including 15,015 men and 16,149 women. Supplementary Figure S1 presents a flowchart of participant selection.

### 2.2. Dietary Assessment

Information regarding food intake was collected using 24 h dietary recall interviews for two non-consecutive days. The first 24 h dietary recall interview was conducted in person, and the second 24 h dietary recall interview was conducted 3–10 days afterwards via telephone. Dietary nutrient and energy values were estimated using the United States Department of Agriculture’s Food and Nutrient Database for Dietary Studies (FNDDS). Based on the two 24 h dietary recall interviews, the mean values of dietary fiber consumption were calculated. We determined three meals according to the name of the meal given by the participants for individual foods and then calculated the amount of dietary fiber in three meals. We defined breakfast as foods selected as ‘breakfast’ or ‘desayao’ (Spanish for breakfast), lunch as foods selected as ‘lunch’ or ‘comida’ (Spanish for lunch), and dinner as foods selected as ‘dinner/supper’ or ‘cena’ (Spanish for dinner).

To reduce extraneous variation, the intake of dietary fiber for energy was determined using regression analysis of the residual method, in which the total dietary fiber intake was corrected for the total energy intake, and the fiber intake during three meals was corrected for the corresponding energy intake per meal [28]. The residual method computed energy-adjusted nutrient intake as the residuals of a regression model of energy intake (independent variable) and the corresponding absolute nutrient intake (dependent variable). This approach isolated the variation in nutrient intake attributed to the composition of the diet from the variation in nutrient intake attributed to the amount of energy consumed and then obtained the nutrient intake independent of energy intake and reflecting dietary composition. An additional strength of the residual method is that the collinearity problem is removed, which occurs when a multivariable approach is used, and energy intake is related to the nutrients of interest. The total dietary fiber intake and the intake of fiber during three meals were adjusted for corresponding energy values with the use of the residual method, before categorizing intake into quintiles.

### 2.3. Main Exposure

The main exposures in this study were the intake of dietary fiber at breakfast, lunch, and dinner via regression analysis of the residual method. The control in different variables was fiber intake in the lowest category in one variable, comparing the highest versus the lowest category.

### 2.4. Main Outcomes

The outcome variable was mortality status, which was determined using the National Death Index (NDI) from 31 December 2015. The NDI is a highly reliable and widely used resource for death identification. The documents related to the NDI mortality can be accessed at https://www.cdc.gov/nchs/data-linkage/mortality.htm (accessed on 11 May 2022), https://www.cdc.gov/nchs/nvss/mortality/lcwk9.htm (accessed on 17 May 2017). We merged the baseline survey data with the 2015 public-use Linked Mortality File (LMF) to assess the survival status of the participants. Information extracted from the LMF included vital status, the number of person–months of follow-up from the NHANES interview date, and the underlying leading cause of death. The International Classification of Diseases (ICD)-10 was used to determine disease-specific deaths. Death due to cancer was defined as I CD-10 codes C00-C97. Death due to CVD was defined as ICD-10 codes I00–I09, I11, I13, I20–I51, or I60–I69. Participants with missing vital statuses were excluded from this study. In total, 2771 deaths, including 631 deaths due to cancer and 836 deaths due to CVD, were documented.

### 2.5. Confounding and Effect Modification Measurements

The non-dietary data included age (years) (divided into five equal groups); gender (male/female); race/ethnicity (non-Hispanic White/non-Hispanic Black/Mexican American/other); education level (<9th grade, 9th–11th grade, high-school graduate, GED or equivalent, some college or Associate in Arts degree, or college graduate or above); annual household income (<USD 20,000, USD 20,000–USD 45,000, USD 45,000–USD 75,000, USD 75,000–USD 100,000, or >USD 100,000), BMI (kg/m^2^); whether the individual exercises regularly (yes/no); whether they are a current smoker (yes/no); whether they are a current drinker (yes/no); whether they had disease history of diabetes, hypertension, and dyslipidemia (yes/no); whether they used medicine for lower blood sugar, hypertension, and cholesterol (yes/no); systolic and diastolic blood pressure (mm/Hg); fasting plasma glucose (mmol/L); HbA1c (%); HDL cholesterol (mmol/L); and triglycerides (mmol/L). The dietary measurement covariates included the total energy (kcal/day) (divided into ten equal groups); energy intake at breakfast, lunch, and dinner; total dietary fiber (low/high); residual fiber intake at breakfast, lunch, and dinner; percentage of energy provided by total protein, by carbohydrates, and by fat; timing variable (eats three meals a day, only skips breakfast, only skips lunch, only skips dinner, only eats breakfast, only eats lunch, or only eats dinner); dietary supplement use (yes/no); and diet quality (divided into ten equal groups), which was calculated using the Alternative Healthy Eating Index (AHEI) [29].

### 2.6. Statistical Analysis

All the statistical analyses were conducted using R 3.6.1 (The R Foundation for Statistical Computing, Vienna, Austria), and a two-sided *p*-value < 0.05 was considered to be statistically significant. Demographic characteristics, nutrient intake, and anthropometric measurements were expressed as relative frequencies. Continuous variables are presented as means and standard deviations (SDs). Categorical variables are presented as numbers and percentages. General linear models after adjusting for age were used to compare baseline characteristics by quintiles of residual total dietary fiber intake and residual dietary fiber intake per meal.

In this study, Cox proportional hazards (CPHs) regression models, widely used to analyze time-to-event data, were developed to evaluate the association between the total dietary fiber intake, fiber intake during three meals, and CVD, cancer, and all-cause mortality rates. In CPH regression models, residual total fiber intake was modeled as quintiles (with the lowest quintile used as a reference), and the residual fiber intakes in three meals were modeled both as continuous variables and quintiles. The survival time was the number of months between the NHANES interview date and death or the end of the follow-up period (31 December 2015). In the analysis of total dietary fiber intake, we calculated hazard ratios (HRs) for models 1 and 2. Covariates in model 1 included age, sex, classification of BMI, ethnicity, income, education level, smoking and drinking status, regular exercise, nutrient supplement use, total intake of energy, percentage of energy provided by protein, fat, and carbohydrates, AHEI, the prevalence of diabetes, hypertension, and hyperlipidemia, and medication use for glucose or blood pressure or blood lipids. Covariates in model 2 were based on model 1 with the additional adjustment of a timing variable. In the analysis of three meals, model 3 was used, which was based on model 1 with additional adjustments for total dietary fiber intake, energy intake at breakfast, lunch, and dinner, and residual fiber intake at breakfast, lunch, and dinner. In this study, the dietary fiber intake in three meals was estimated using the same model. To test the linear trend, we modeled categorical variables as continuous by assigning the median value to each quintile.

In nutritional epidemiology, food substitution models have been used to study the relationship between nutrient or food substitution and related health or disease outcomes and offer dietary advice for the prevention and treatment of diseases [30]. Today, many studies have recognized and used the method of substitution analysis to study the substitution of one food or nutrient with another under the premise of equal energy or equal intake, and then observed changes in epidemiological indicators [5,31,32]. Based on the CPH model mentioned above, we established an equivalent dietary fiber substitution model to evaluate the changes in all-cause and cancer mortality risks caused by switching dietary fiber intake from a single time period to another single time period. In this study, we used substitution analyses to determine whether replacing each SD of residual fiber intake at breakfast or lunch with dinner was associated with a variation in all-cause, cancer, and CVD mortality risks. The standard deviation standardization method was used to process the data regarding residual fiber intake during three meals in order to eliminate the effect of unit dimension. The processed data conformed to the standard normal distribution with the mean value of 0 and SD value of 1.

### 2.7. Sensitivity Analysis

Three sets of sensitivity analyses were performed in this study. In set 1, the association between the difference in dietary fiber intake at dinner versus breakfast or lunch and all-cause, cancer, and CVD mortalities was determined to examine whether difference between the amount of fiber intake in two meals could provide more information than the amount of fiber intake in a single meal. In set 2, we excluded the participants who did not eat breakfast to eliminate the effect of skipping breakfast. In set 3, we corrected the total vegetable and fruit intake at breakfast, lunch, and dinner to eliminate the effect of differences in their intake in three meals.

## 3. Results

### 3.1. Baseline Characteristics

Table 1 presents the demographic and nutrition characteristics in terms of residual total dietary fiber intake in quintiles. Compared with those in quintiles 1–4, participants in quintile 5 were more likely to be older, women, exercise regularly, eat three meals a day, have higher incomes and education levels, have higher total dietary fiber intakes, score better in the AHEI, use dietary supplements, control blood sugar, blood pressure and cholesterol, have a higher prevalence of diabetes, hypertension, and dyslipidemia, and have higher contributive rates of protein and carbohydrates in total energy (*p* < 0.01), but they were less likely to be current smokers and drinkers, with a lower non-Hispanic White ratio, BMI, total energy consumption, and lower contributive rate of fat in total energy (*p* < 0.01). Table 2 presents the nutrition characteristics in terms of residual fiber intake per meal in quintiles. Compared with those in quintiles 1–4, participants in quintile 5 were more likely to have higher dietary fiber intake at breakfast, lunch, and dinner (*p* < 0.01).

### 3.2. Cox Proportional Hazards Models

**Figure 1:**
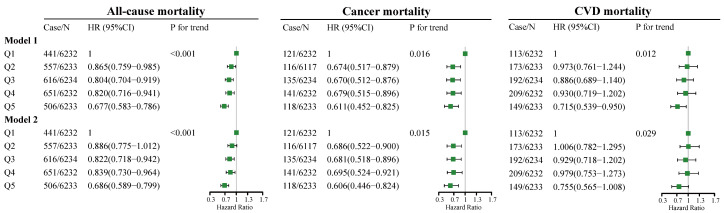
Adjusted HRs for all-cause, cancer, and CVD mortality by quintiles of total dietary fiber intake. Model 1 adjustments include age (5 categories); sex; classification of BMI; ethnicity, income; education level; smoking and drinking status; regular exercise; nutrient supplement use; total intake of energy (10 categories); percentage of energy provided by protein, fat, and carbohydrates; AHEI (10 categories); the prevalence of diabetes, hypertension, and hyperlipidemia; and medication use for glucose or blood pressure or blood lipids. Model 2 is based on model 1, with the additional adjustment of a timing variable. BMI, body mass index; AHEI, Alternative Healthy Eating Index; CVD, cardiovascular disease; Case/*n*, number of case participants/total; Q, Quintile; HR, hazard ratio.

The association between total dietary fiber intake and the all-cause, cancer, and CVD mortality rate is presented in Figure 1. In model 1, as indicated by HR and 95% CI values, compared to the participants in the lowest quintile of total fiber intake (quintile 1), participants in the highest quintile of total fiber intake (quintile 5) had lower all-cause (HR = 0.677, 95% CI: 0.583–0.786, *p* for trend < 0.001), cancer (HR = 0.611, 95% CI: 0.452–0.825, *p* for trend = 0.016), and CVD (HR = 0.715, 95% CI: 0.539–0.950, *p* for trend = 0.012) mortality risks. In model 2, compared to the participants in the lowest quintile of total fiber intake, participants in the highest quintile of total fiber intake had lower all-cause (HR = 0.686, 95% CI: 0.589–0.799, *p* for trend <0.001) and cancer (HR = 0.606, 95% CI: 0.446–0.824, *p* for trend <0.015) mortality risks, but no significant association between total fiber intake and CVD mortality risk was found.

**Figure 2:**
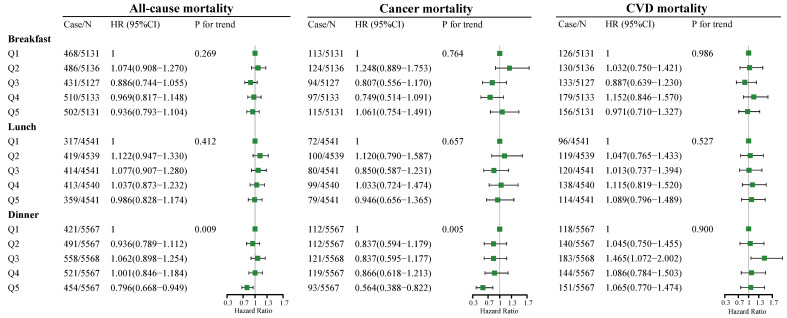
Multivariate adjusted HRs of dietary fiber intake at breakfast, lunch, and dinner with all-cause, cancer, and CVD mortality. Adjusted covariates in model 3 are based on model 1 with additional adjustment of total dietary fiber intake(low/high), energy intake at breakfast, lunch, and dinner, and residual fiber intake at breakfast, lunch, and dinner. Covariates in model 1 include age (5 categories); sex; classification of BMI; ethnicity; income; education level; smoking and drinking status; regular exercise; nutrient supplement use; total intake of energy (10 categories); percentage of energy provided by protein, fat, and carbohydrates; AHEI (10 categories); the prevalence of diabetes, hypertension, and hyperlipidemia; and medication use for glucose or blood pressure or blood lipids. BMI, body mass index; AHEI, Alternative Healthy Eating Index; CVD, cardiovascular disease; Case/*n*, number of case participants/total; Q, Quintile; HR, hazard ratio.

The association between dietary fiber intake at breakfast, lunch, and dinner and all-cause, cancer, and CVD mortality risks is presented in Figure 2. At breakfast and lunch (Figure 2), no significant association between fiber intake and all-cause, cancer, and CVD mortality risks was observed. At dinner (Figure 2), as indicated by HR and 95% CI values, compared to the participants in the lowest quintile of fiber intake (quintile 1), participants in the highest quintile of fiber intake (quintile 5) had lower all-cause (HR = 0.796, 95% CI: 0.668–0.949, *p* for trend = 0.009) and cancer (HR = 0.564, 95% CI: 0.388–0.822, *p* for trend = 0.005) mortality risks, but no significant association between fiber intake and CVD mortality risk was found.

The adjusted HRs and coefficients for the all-cause, cancer, and CVD mortality risks per 1 SD difference in fiber intake during three meals are presented in Supplementary Table S1. At dinner (Supplementary Table S1), after adjustment for potential confounders, we found a statistically significant linear inverse association between all-cause and cancer mortality risks and residual fiber intake (coefficient_all-cause_ = −0.063, HR_all-cause_ = 0.939, 95% CI: 0.883–0.988; coefficient_cancer_ = −0.160, HR_cancer_ = 0.852, 95% CI: 0.746–0.972 with an increase per 1 SD in residual dietary fiber at dinner) and a statistically insignificant linear association between CVD mortality risk and residual fiber intake. At breakfast and lunch (Supplementary Table S1), after adjusting for potential confounders, we found a statistically insignificant linear association between all-cause, cancer, and CVD mortality risks and residual fiber intake. 

### 3.3. Equivalent Substitution Analysis

**Figure 3:**
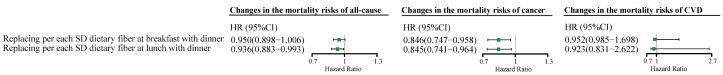
Multivariate adjusted HRs for all-cause, cancer, and CVD mortality: equivalent substitution of dietary fiber from breakfast and lunch to dinner. Adjusted covariates in model 3 are based on model 1 with additional adjustment of total dietary fiber intake(low/high), energy intake at breakfast, lunch, and dinner, and residual fiber intake at breakfast, lunch, and dinner. Covariates in model 1 include age (5 categories); sex; classification of BMI; ethnicity; income; education level; smoking and drinking status; regular exercise; nutrient supplement use; total intake of energy (10 categories); percentage of energy provided by protein, fat, and carbohydrates; AHEI (10 categories); the prevalence of diabetes, hypertension, and hyperlipidemia; and medication use for glucose or blood pressure or blood lipids. BMI, body mass index; AHEI, Alternative Healthy Eating Index; CVD, cardiovascular disease; Case/*n*, number of case participants/total; Q, Quintile; HR, hazard ratio.

Figure 3 shows changes in the all-cause, cancer, and CVD mortality risks in predicted equivalent substitution models by replacing each SD of dietary fiber intake at breakfast and lunch with dinner, respectively.

The results show that the HRs for cancer mortality decreases by 15.4% (HR = 0.846, 95% CI: 0.747–0.958) in models with each SD of dietary fiber intake at breakfast being equivalently switched to dinner. Likewise, the results show HRs for all-cause mortality reduces by 6.4% (HR = 0.936, 95% CI: 0.883–0.993) and for cancer mortality by 15.5% (HR = 0.845, 95% CI: 0.741–0.964) in models with each SD of dietary fiber intake at lunch being equivalently switched to dinner. Additionally, there were no significant associations between CVD mortality and each SD of dietary fiber intake at breakfast being equivalently switched to dinner or lunch.

### 3.4. Sensitivity Analysis

In the first set of sensitivity analyses, at dinner versus breakfast, compared to the participants in the lowest quintile of difference in dietary fiber intake, participants in the highest quintile of difference in dietary fiber intake had lower all-cause and cancer mortality risks, but no significant association between difference in dietary fiber intake and CVD mortality risk was found (Supplementary Figure S2). At dinner versus lunch, no significant association was found between the difference in fiber intake and all-cause, cancer, and CVD mortality risks (Supplementary Figure S3). These results suggest that the difference in the amount of fiber intake between two meals could provide more information than the amount of fiber intake in one meal alone. In the second set of analyses, after excluding those who skipped breakfast, the association between fiber intake at dinner and mortality outcomes was still significant, but no significant association between fiber intake and mortality outcomes was observed at breakfast and lunch (Supplementary Table S2). In the third set of analyses, after correcting the total vegetable and fruit intake in three meals, the association between fiber intake at dinner and all-cause and cancer mortality outcomes was still significant, but no significant association between fiber intake and mortality outcomes was observed at breakfast and lunch (Supplementary Table S3).

## 4. Discussion

The results of analyses regarding data from the NHANES 2003–2014 indicate that higher dietary fiber intake is associated with decreased all-cause and cancer mortality risks, but this is not found for CVD mortality risk. Fiber intake at dinner was associated with decreased cancer and all-cause mortality risks, but this was not found for CVD mortality risk, whereas fiber intake at breakfast and lunch was not significantly associated with all-cause and specific disease mortality risks. Furthermore, substituting each SD intake of dietary fiber at breakfast with dinner markedly reduced the cancer mortality risk by 15.4%.

To the best of our knowledge, this is the first study to examine the association between dietary fiber intake in three meals across the day and all-cause, cancer, and CVD mortality risks. Our study supplied evidence to support the benefits of high dietary fiber intake at dinner and highlighted the importance of dietary fiber intake time in reducing the risks of all-cause and cancer mortality. Dietary fiber is a key element in healthy eating and plays an important role in the occurrence and development of many chronic diseases [33].

One of the findings in this study supported a significant association between higher total dietary fiber intake and lower all-cause and cancer mortality risks after multivariable adjustment, regardless of whether the timing variable was included in the model. The association was significant for CVD mortality in the analysis of the multivariable-adjusted model, but once the confounding factor of the timing variable was included in the model, the association between fiber intake and CVD mortality was not significant. Similarly, a systematic review and meta-analysis of cohort studies in which the model did not include the timing variable found that participants with higher fiber intake had lower cancer, all-cause, and CVD mortality rates (HR_cancer_: 0.83, 95% CI: 0.74–0.91; HR_all-cause_: 0.77, 95% CI: 0.73–0.81; HR_CVD_: 0.77, 95% CI: 0.72–0.81) [10]. The inconsistent association between fiber intake and CVD mortality risk in different models is likely due to the effect of the number of eating occasions/eating frequency. A scientific statement from the American Heart Association showed that eating patterns that focus on eating occasions/eating frequency could affect cardiometabolic health [34]. A cohort study from the NHANES (1999–2002) showed a significant association between eating breakfast and lower all-cause and CVD mortality rates and the significant role of fiber intake; there was no association between fiber and mortality rates in non-breakfast eaters. In addition, this study also showed that breakfast eaters with high daily total fiber intake had lower all-cause mortality risks compared to their counterparts with low fiber intake, but this association was not significant for CVD mortality risk [35]. This indicates that breakfast-eating status has an important role in the association between fiber intake and mortality risk. Other meta-analyses of observational studies that examined the risks of esophageal cancer and breast cancer found that the pooled odds ratio (OR) or relative risk (RR) was lower for individuals with higher fiber intake (comparing the highest versus the lowest category, OR_esophageal cancer_: 0.52, 95% CI: 0.43–0.64; RR_breast cancer_: 0.92, 95% CI: 0.88–0.95) [11,12]. These systematic reviews included dozens of studies and were supportive of the association between fiber intake and all-cause and cancer mortality. Compared with those studies, our study regarding total dietary fiber was more comprehensive, as it not only included more covariates in models, but also had a wider age range, and in particular, extra adjusted AHEI and timing variables were included in models. Furthermore, we originally analyzed the relationship between fiber intake and mortality at different time periods, which they did not.

The most meaningful finding in this study was that a higher intake of dietary fiber at dinner was significantly associated with lower all-cause and cancer mortality risks, though the same association was not found for CVD mortality risk, whereas dietary fiber consumed at breakfast and lunch did not have these beneficial effects. Studies have demonstrated that there are clock genes in the gastrointestinal tract that regulate its activity and function [36]. A randomized controlled trial of 16 people showed that, for evening meals, the rate of human gastric emptying was considerably slower than that for morning meals [37]. In addition, it was shown that the consumption of dietary fiber causes fermentation via colonic microbiota, which produce short-chain fatty acids. This was shown to increase the viscosity of stomach contents, slow down the rate of gastric emptying, and prolong glucose absorption, which could slow the rate of the absorption of glucose molecules into the intestine, improve insulin function, and lower glycemic loads [38,39]. Therefore, the intake of more dietary fiber at dinner agrees with biological rhythms. A previous study suggested that young adult insulin sensitivity was responsive to higher evening, but not morning intakes of higher glycemic index carbohydrates. Postprandial blood glucose increases caused by foods containing higher glycemic index carbohydrates are particularly harmful in the evening and may have long-term adverse effects on adult metabolic health and increase the risk of diabetes [40], which is associated with an increased incidence of and mortality from many cancers [41]. Hence, the intake of more dietary fiber at dinner slows down the rate of gastric emptying and helps maintain a low glycemic load in the evening, which is better for human health.

Moreover, dietary fiber could be fermented by intestinal flora, producing short-chain fatty acids (SCFAs), which have a vital role in maintaining intestinal immune homeostasis and protecting against inflammation and carcinogenesis [42,43]. In healthy human bodies, Firmicutes and Bacteroidetes make up more than 90% of the gastrointestinal bacteria [44]. Compared with healthy people, it was shown that the proportion of Firmicutes in obese individuals increased, while the proportion of Bacteroidetes decreased, indicating that an increase in Firmicutes is related to obesity, and an increase in Bacteroidetes is more beneficial to health [45]. Additionally, most studies support the claim that an increase in Firmicutes contributes to the development of obesity [46]. The abundance of intestinal flora in humans and animals oscillates the circadian rhythm and is involved in host metabolic regulation [47]. Studies have shown that various bacterial groups with a relative abundance of about 15% show circadian rhythm oscillations, including the orders Clostridiales, Lactobacillales, and Bacteroidales, accounting for 60% of the total intestinal flora [47]. In the absolute abundance of gut microbiota in mice, Bacteroidetes oscillate diurnally and Firmicutes only showed slight fluctuations over time [48]. However, in the relative abundance of intestinal microbiota in mice, Bacteroidetes reached their peak during the dark phase and a low point during the light phase; in contrast, Firmicutes peaked around the beginning of the light phase and fell during the dark phase [24]. This could be attributable to the high abundance and oscillation of Bacteroidetes, which produce significant variation, which is the main driving force of the circadian oscillation of total bacterial load. Firmicutes remain relatively stable in a day, and their proportion changes in proportion to changes in levels of Bacteroidetes [48]. Low fiber intake in mice was shown to induce an increase in Firmicutes and a decrease in Bacteroidetes, and a similar effect has been observed in humans [49,50]. A randomized controlled study in healthy subjects showed that the dietary fiber in a tested dinner of rye kernel bread has anti-diabetic potential and could be beneficial in the prevention of obesity, which could probably be mediated by colonic fermentation, increasing next-morning gut fermentation activity, which is indicated by increased plasma short-chain fatty acid concentrations [51]. This may indicate that higher dietary fiber intake at dinner increases the abundance of Bacteroidetes, which also conforms to the circadian rhythm of intestinal flora and reduces the risk of obesity, a risk factor of cancer, which would aid the maintenance of better health.

In addition, a high-fiber diet potentially lowers inflammation by modifying both the pH and the permeability of the gut [52]. In mice, fiber reduced the expression of genes encoding inflammatory cytokines, chemokines, and fibrosis-promoting proteins in diabetic kidneys [53]. It has been reported that pro-inflammatory cytokines had an internal circadian pattern as well, showing the lowest in the afternoon and gradually increasing during the night [54]. Therefore, a higher intake of dietary fiber at dinner could be more likely to decrease the amplitude of inflammation at night and probably reduces the cancer mortality risk.

## 5. Strengths and Limitations

This study has several strengths. Firstly, this was the first study to examine the association between dietary fiber intake time across the day and cancer, CVD, and all-cause mortality risks. Our findings emphasized that the core of chrono-nutrition, when you eat is as important as what you eat. The consumption time of dietary fiber should coordinate with body clock fluctuations and match the body’s metabolic rhythm to reduce the cancer mortality risk. Secondly, the NHANES is a nationally representative database based on a probability sample survey design in the U.S., which provides the most comprehensive and authoritative dietary intake studies as well as a detailed evaluation of lifestyle factors. However, several certain limitations also should be considered. First, 24 h dietary recall methods, the most valid and commonly used instrument in observational studies, were used to collect dietary consumption information, but we cannot exclude the possibility of dietary measurement errors. Second, we used two dietary measurements within two weeks to predict the future diet and lifestyle of the current population; this assumes that dietary practices remain similar over time. Hence, further studies are necessary to demonstrate the conclusions using cohorts including people of different races or from different countries. Third, this study was not able to distinguish different types of dietary fiber. Thus, in further studies, it will be necessary to determine the long-term effects of dietary fiber intake, including different types of fiber across meals, on cancer and all-cause mortality outcomes. Fourth, we used substitution analyses to determine whether replacing each SD of residual fiber intake at breakfast or lunch with dinner was associated with a variation in mortality risks. However, we cannot be sure whether the substitution could be accomplished by dietary changes including dietary fiber. Fifth, we used CPH regression models to determine whether quintiles of fiber intake at each meal were associated with a variation in mortality risks while a high-quintile-to-low-quintile contrast in fiber intake is different across meals. Therefore, it would be better to compare fiber intake for each meal instead of quintiles, which could make the results more direct, while finding a fit model to directly compare fiber intake between each meal is a challenge. Finally, our analysis did not use the NHANES sampling parameters. Therefore, our conclusions only apply to the NHANES (2003–2014) cohort study population and cannot be generalizable to the U.S. population as a whole.

## 6. Conclusions

Chrono-nutrition, which combines nutritional research and chrono-biology, emphasizes the impact of the timing of eating, including dietary fiber intake, on health outcomes. This study provides evidence that can be used to guide and improve dietary guidelines and individual precision nutrition. In conclusion, this study demonstrated that, for the NHANES (2003–2014) cohort, to achieve reductions in all-cause and cancer mortality risks, the optimal intake time of dietary fiber was in the evening.

## Figures and Tables

**Table 1:** Baseline characteristics of participants according to quintiles of total dietary fiber intake by the residual method among adults in the NHANES, 2003–2014.

Variables	Quintile 1 (*n* = 6232)	Quintile 2 (*n* = 6233)	Quintile 3 (*n* = 6234)	Quintile 4 (*n* = 6232)	Quintile 5 (*n* = 6233)	*p*-Value
Age, years	39.51 (16.97)	45.25 (19.47)	48.99 (19.43)	51.29 (19.00)	51.39 (18.10)	<0.001
Female, %	2506 (40.21)	3477 (55.78)	3604 (57.81)	3511 (56.34)	3051 (48.95)	<0.001
Non-Hispanic White, %	2807 (45.04)	2832 (45.44)	2986 (47.90)	2920 (46.85)	2691 (43.17)	<0.001
BMI, kg/m^2^	28.95 (7.45)	29.04 (7.12)	28.97 (6.91)	28.72 (6.45)	28.12 (6.08)	<0.001
College graduate or above, %	652 (10.46)	943 (15.13)	1235 (19.81)	1545 (24.79)	1928 (30.93)	<0.001
>USD 100,000 annual household income, %	418 (6.71)	544 (8.73)	624 (10.01)	729 (11.70)	912 (14.63)	0.002
Exercise regularly, %	1379 (22.13)	1399 (22.45)	1421 (22.79)	1436 (23.04)	1657 (26.58)	<0.001
Current smoker, %	2663 (42.73)	1771 (28.41)	1290 (20.69)	928 (14.89)	718 (11.52)	<0.001
Current drinker, %	4192 (67.27)	3863 (61.98)	3885 (62.32)	3852 (61.81)	3976 (63.79)	<0.001
Dietary supplements use, %	2020 (32.41)	2693 (43.21)	3143 (50.42)	3467 (55.63)	3665 (58.80)	<0.001
Diabetes, %	571 (9.28)	865 (14.08)	964 (15.72)	1119 (18.20)	1005 (16.40)	<0.001
Hypertension, %	2851 (45.76)	3100 (49.75)	3207 (51.44)	3266 (52.42)	3153 (50.59)	<0.001
Dyslipidemia, %	2008 (32.22)	2316 (37.16)	2565 (41.15)	2696 (43.26)	2751 (44.14)	<0.001
Medicine use for lower blood sugar, %	1321 (21.20)	1448 (23.23)	1615 (25.91)	1676 (26.89)	1506 (24.16)	<0.001
Medicine use for hypertension, %	1114 (17.88)	1561 (25.04)	1770 (28.39)	1805 (28.96)	1712 (27.47)	<0.001
Medicine use for lower cholesterol, %	874 (14.02)	1263 (20.26)	1585 (25.43)	1694 (27.18)	1651 (26.49)	<0.001
Total energy, kcal/day	2306.22 (826.46)	1883.70 (740.46)	1858.92 (718.60)	1930.49 (706.57)	2161.03 (737.03)	<0.001
Total fat, % of energy	33.92 (8.33)	34.47 (7.28)	33.53 (7.22)	32.65 (7.14)	30.92 (7.45)	<0.001
Carbohydrate, % of energy	46.76 (11.31)	48.29 (9.32)	49.71 (8.76)	50.80 (8.48)	53.59 (8.69)	<0.001
Protein, % of energy	15.13 (4.71)	15.78 (4.39)	16.12 (4.23	16.40 (4.19)	16.36 (3.91)	<0.001
Total dietary fiber, g/day	9.18 (4.17)	11.16 (4.22)	13.99 (4.14)	18.08 (4.23)	28.12 (8.63)	<0.001
AHEI	51.82 (13.70)	48.91 (13.90)	50.42 (13.26)	53.06 (12.81)	56.84 (12.42)	<0.001
Three meals a day, %	2588 (42.25)	3098 (50.41)	3525 (56.95)	3930 (63.45)	4165 (67.10)	<0.001
Only breakfast skipping, %	916 (14.96)	738 (12.01)	572 (9.24)	386 (6.23)	283 (4.56)	<0.001
Only lunch skipping, %	1255 (20.49)	1223 (19.90)	1214 (19.61)	1129 (18.23)	1093 (17.61)	<0.001
Only dinner skipping, %	356 (5.81)	389 (6.33)	379 (6.12)	379 (6.12)	379 (6.12)	<0.001
Only eat breakfast, %	138 (2.25)	121 (1.97)	109 (1.76)	81 (1.31)	82 (1.32)	<0.001
Only eat lunch, %	230 (3.76)	156 (2.54)	102(1.65)	83 (1.34)	48 (0.77)	<0.001
Only eat dinner, %	642 (10.48)	421 (6.85)	289(4.67)	206 (3.33)	157 (2.53)	<0.001

Continuous variables are presented as mean and standard derivation (SD). Categorical variables are presented as numbers and percentages. *p*-values are calculated by a general linear model for variables adjusting for age. Hypertension is defined by a self-reported diagnosis, the systolic blood pressure >80 mm Hg, or the diastolic blood pressure >130 mm Hg. Dyslipidemia is defined as serum triglyceride >2.26 mmol/L, or serum cholesterol > 6.22 mmol/L, or low-density lipoprotein >4.14 mmol/L. Diabetes is defined by a self-reported diagnosis, an HbA1c level ≥6.5%, or a fasting plasma glucose level ≥7.0 mmol/L. BMI, body mass index; AHEI, Alternative Healthy Eating Index.

**Table 2:** Baseline characteristics of participants according to quintiles of dietary fiber intake per meal by using the residual method among adults in the NHANES, 2003–2014.

Variables	Total Dietary Fiber Intake per Meal		Residual Dietary Fiber Intake per Meal										
			Quintile 1		Quintile 2		Quintile 3		Quintile 4		Quintile 5		*p*-Value
	*n*	Mean (SD)	*n*	Mean (SD)	*n*	Mean (SD)	*n*	Mean (SD)	*n*	Mean (SD)	*n*	Mean (SD)	
Fiber intake at breakfast, g/day	25,881	3.88 (3.49)	5131	2.40 (1.97)	5136	1.98 (1.78)	5127	2.53 (1.97)	5133	4.12 (2.06)	5131	8.40 (4.21)	<0.001
Fiber intake at lunch, g/day	22,714	5.13 (3.95)	4541	3.60 (2.51)	4539	3.07 (2.25)	4541	3.65 (2.38)	4540	5.25 (2.43	4541	10.08 (2.65)	<0.001
Fiber intake at dinner, g/day	28,072	6.36 (4.68)	5567	4.17 (2.95)	5567	3.824 (2.78)	5568	4.87 (2.86)	5567	6.82 (2.91)	5567	12.18 (5.36)	<0.001
Residual fiber intake at breakfast, g/day	25,658	4.11 (2.65)	5131	1.27 (1.09)	5136	2.85 (0.26)	5127	3.68 (0.25)	5133	4.80 (0.41)	5131	7.97 (2.92)	<0.001
Residual fiber intake at lunch, g/day	22,702	5.06 (2.84)	4541	1.98 (1.20)	4539	3.69 (0.28)	4541	4.59 (0.27)	4540	5.77 (0.45)	4541	9.29 (2.93)	<0.001
Residual fiber intake at dinner, g/day	27,836	6.53 (3.39)	5567	2.80 (1.52)	5567	4.85 (0.33)	5568	5.98 (0.35)	5567	7.47 (0.56)	5567	11.53 (3.43)	<0.001

Continuous variables are presented as mean and standard derivation (SD). *p*-values are calculated by general linear model for variables adjusting for age.

## Data Availability

The data presented in this study are openly available in the U.S. national health and nutrition examination survey at [https://www.cdc.gov/nchs/nhanes/index.htm], reference number 2003–2014.

## Supplementary Materials

The following are available online at https://www.mdpi.com/article/10.3390/nu14122521/s1, Figure S1: Flowchart of participant selection according to NHANES surveys conducted during the period 2003–2014. Figure S2: Adjusted HRs for the differences in dietary fiber intake at dinner versus breakfast and all-cause, cancer, and CVD mortality. Figure S3: Adjusted HRs for the differences in dietary fiber intake at dinner versus lunch and all-cause, cancer, and CVD mortality. Table S1: Adjusted HRs and coefficients for the mortality of all-cause, cancer, and CVD by per 1 SD difference in fiber intake at three meals. Table S2: Adjusted HRs of dietary fiber intake at breakfast, lunch, dinner, and all-cause, cancer, and CVD mortality with additionally adjusted for breakfast skipping. Table S3: Adjusted HRs of dietary fiber intake at breakfast, lunch, dinner and all-cause, cancer, and CVD mortality with additionally adjusted for total vegetable and fruit intake at breakfast, lunch, and dinner.
