# Supplementary material for: The Association of Dietary Fiber Intake in Three Meals with All-Cause and Disease-Specific Mortality among Adults: The U.S. National Health and Nutrition Examination Survey, 2003–2014
Source: Nutrients. 2022 Jun 17;14(12):2521. doi: 10.3390/nu14122521 (PMC9228910; doi:10.3390/nu14122521)
Supplement: Supplementary file 1 [file nutrients-14-02521-s001.zip › nutrients-1716913-supplementary.pdf]

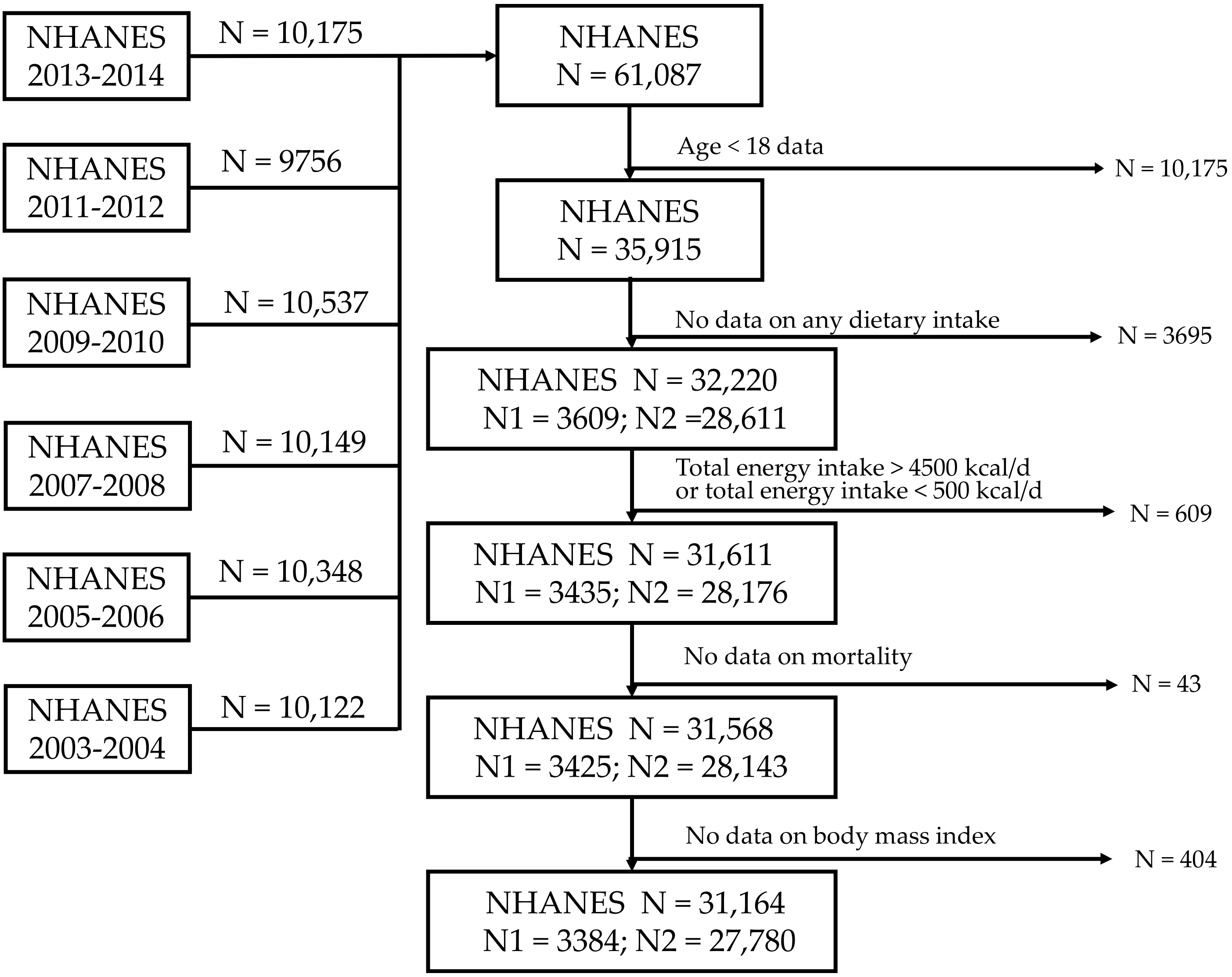

Supplementary Figure S1. Flowchart of participant selection according to NHANES surveys conducted during the period 2003–2014. N, number of total participants; N1, number of participants who only had one-day food intake information; N2, number of participants who had two-day food intake information.

	All-cause mortality			Cancer mortality			CVD mortality		
	Case/N	HR (95%CI)	P for trend	Case/N	HR (95%CI)	P for trend	Case/N	HR (95%CI)	P for trend
**Dinner versus breakfast**									
Q1	498/4649	1	0.002	125/4649	1	0.001	145/4649	1	0.001
Q2	479/4650	0.918(0.792−1.063)		101/4650	0.658(0.481−0.902)		146/4650	0.658(0.481−0.902)	
Q3	426/4649	0.882(0.755−1.030)		97/4649	0.704(0.512−0.967)		128/4649	0.704(0.512−0.967)	
Q4	413/4650	0.886(0.760−1.033)		100/4650	0.805(0.592−1.094)		120/4650	0.805(0.592−1.094)	
Q5	349/4649	0.763(0.647−0.900)		72/4649	0.485(0.336−0.699)		115/4649	0.485(0.336−0.699)	

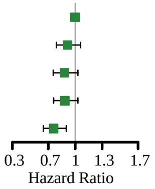

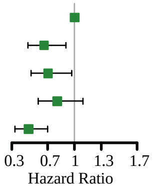

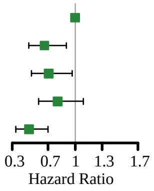

Supplementary Figure S2. Adjusted HRs for the differences in dietary fiber intake at dinner versus breakfast and all-cause, cancer, and CVD mortality. Adjustments included age (5 categories), sex, classification of BMI, ethnicity, income, education level, smoking and drinking status, regular exercise, nutrient supplement use, percentage of energy provided by protein, fat, and carbohydrates, AHEI (10 categories), the prevalence of diabetes, hypertension, and hyperlipidemia, and medication use for glucose or blood pressure or blood lipids, total intake of energy (10 categories), total intake of dietary fiber (low/high), the sum of energy for breakfast and dinner, energy intake at lunch, residual fiber intake at lunch. BMI, body mass index; AHEI, Alternative Healthy Eating Index; CVD, cardiovascular disease; Case/N, number of case participants/total; Q, Quintile; HR, hazard ratio.

	All-cause mortality			Cancer mortality			CVD mortality		
	Case/N	HR (95%CI)	P for trend	Case/N	HR (95%CI)	P for trend	Case/N	HR (95%CI)	P for trend
**Dinner versus lunch**									
Q1	312/4032	1	0.281	72/4032	1	0.277	95/4032	1	0.738
Q2	364/4047	1.142(0.974−1.338)		77/4047	1.000(0.716−5.338)		115/4047	1.167(0.874−7.338)	
Q3	347/4054	1.035(0.879−3.338)		76/4054	0.904(0.642−3.338)		105/4054	1.083(0.804−9.338)	
Q4	333/4045	0.954(0.811−6.338)		80/4045	0.905(0.648−8.338)		98/4045	0.921(0.684−4.338)	
Q5	332/4028	0.977(0.831−8.338)		73/4028	0.848(0.602−7.338)		103/4028	1.041(0.777−5.338)	

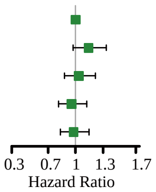

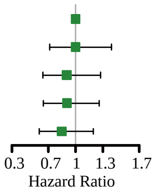

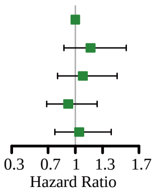

Supplementary Figure S3. Adjusted HRs for the differences in dietary fiber intake at dinner versus lunch and all-cause, cancer, and CVD mortality. Adjustments included age (5 categories), sex, classification of BMI, ethnicity, income, education level, smoking and drinking status, regular exercise, nutrient supplement use, percentage of energy provided by protein, fat, and carbohydrates, AHEI (10 categories), the prevalence of diabetes, hypertension, and hyperlipidemia, and medication use for glucose or blood pressure or blood lipids, total intake of energy (10 categories), total intake of dietary fiber (low/high), the sum of energy for lunch and dinner, energy intake at breakfast, residual fiber intake at breakfast. BMI, body mass index; AHEI, Alternative Healthy Eating Index; CVD, cardiovascular disease; Case/N, number of case participants/total; Q, Quintile; HR, hazard ratio.

Supplementary Table S1. Adjusted HRs and coefficients for the mortality of all-cause, cancer, and CVD by per 1 SD difference in fiber intake at three meals.

Residual fiber continuous [a]	All-cause mortality			Cancer mortality			CVD mortality		
	Case	Coefficient	HR (95% CI)	Case	Coefficient	HR (95% CI)	Case	Coefficient	HR (95% CI)
Breakfast	2397	-0.017	0.983 (0.931-1.039)	543	0.007	1.007 (0.899-1.129)	724	0.023	1.023 (0.932-1.124)
Lunch	1922	0.004	0.893 (1.004-0.948)	430	0.011	1.011 (0.895-1.142)	587	0.053	1.054 (0.954-1.164)
Dinner	2445	-0.063	0.939 (0.883-0.998)	557	-0.160	0.852 (0.746-0.972)	736	-0.028	0.972 (0.871-1.085)

Adjustments included age (5 categories), sex, classification of BMI, ethnicity, income, education level, smoking and drinking status, regular exercise, nutrient supplement use, percentage of energy provided by protein, fat, and carbohydrates, AHEI (10 categories), the prevalence of diabetes, hypertension, and hyperlipidemia, and medication use for glucose or blood pressure or blood lipids, total intake of energy (10 categories), total intake of dietary fiber (low/high), energy intake at breakfast, lunch and dinner, residual fiber intake at breakfast, lunch and dinner. BMI, body mass index; AHEI, Alternative Healthy Eating Index; CVD, cardiovascular disease; Case, number of case participants; Q, Quintile; HR, hazard ratio; [a] HRs and coefficients for residual dietary fiber continuous at breakfast, lunch, and dinner is each SD.

Supplementary Table S2. Adjusted HRs of dietary fiber intake at breakfast, lunch, dinner, and all-cause, cancer and CVD mortality with additionally adjusted for breakfast skipping.

Residual dietary fiber intake	All-cause mortality		Cancer mortality		CVD mortality	
	Case/N	HR (95% CI)	Case/N	HR (95% CI)	Case/N	HR (95% CI)
**Breakfast**						
Q1	463/5104	1	111/5104	1	126/5104	1
Q2	490/5147	1.105 (0.934-1.307)	126/5147	1.278 (0.909-1.796)	130/5147	1.039 (0.755-1.429)
Q3	436/5154	0.892 (0.749-1.063)	95/5154	0.824 (0.568-1.196)	134/5154	0.881 (0.636-1.222)
Q4	514/5140	0.975 (0.823-1.156)	97/5140	0.747 (0.512-1.090)	181/5140	1.136 (0.834-1.549)
Q5	494/5088	0.936 (0.792-1.107)	114/5088	1.080 (0.766-1.523)	153/5088	0.960 (0.700-1.315)
P for trend		0.216		0.786		0.932
**Lunch**						
Q1	308/3822	1	72/3822	1	96/3822	1
Q2	394/3864	1.039 (0.755-1.429)	91/3864	1.060 (0.752-1.493)	111/3864	1.007 (0.743-1.366)
Q3	384/3545	0.881 (0.636-1.222)	74/3845	0.870 (0.607-1.249)	109/3845	0.982 (0.720-1.340)
Q4	364/3839	1.136 (0.834-1.549)	86/3839	1.012 (0.712-1.439)	123/3839	1.088 (0.803-1.473)
Q5	322/3818	0.960 (0.700-1.315)	72/3818	0.969 (0.674-1.392)	102/3818	1.106 (0.814-1.503)
P for trend		0.622		0.824		0.398
**Dinner**						
Q1	393/4652	1	105/4652	1	107/4652	1
Q2	426/4661	0.939 (0.794-1.111)	89/4661	0.769 (0.548-1.081)	121/4661	1.020 (0.739-1.407)
Q3	496/4638	1.080 (0.916-1.273)	115/4638	0.889 (0.638-1.239)	162/4638	1.430 (1.053-1.943)
Q4	466/4637	1.000 (0.847-1.182)	106/4637	0.820 (0.586-1.148)	134/4637	1.076 (0.782-1.481)
Q5	384/4637	0.790 (0.663-0.941)	80/4637	0.552 (0.379-0.803)	130/4637	1.025 (0.743-1.413)
P for trend		0.007		0.004		0.800

Adjustments included age (5 categories), sex, classification of BMI, ethnicity, income, education level, smoking and drinking status, regular exercise, nutrient supplement use, percentage of energy provided by protein, fat, and carbohydrates, AHEI (10 categories), the prevalence of diabetes, hypertension, and hyperlipidemia, and medication use for glucose or blood pressure or blood lipids, total intake of energy (10 categories), total intake of dietary fiber (low/high), energy intake at breakfast, lunch and dinner, residual fiber intake at breakfast, lunch and dinner. BMI, body mass index; AHEI, Alternative Healthy Eating Index; CVD, cardiovascular disease; Case/N, number of case participants/total; Q, Quintile; HR, hazard ratio.

Supplementary Table S3. Adjusted HRs of dietary fiber intake at breakfast, lunch, dinner and all-cause, cancer and CVD mortality with additionally adjusted for total vegetable and fruit intake at breakfast, lunch and dinner.

Residual dietary fiber intake	All-cause mortality		Cancer mortality		CVD mortality	
	Case/N	HR (95% CI)	Case/N	HR (95% CI)	Case/N	HR (95% CI)
**Breakfast**						
Q1	468/5131	1	113/5131	1	126/5131	1
Q2	486/5136	1.074 (0.908-1.270)	124/5136	1.263 (0.899-1.775)	130/5136	1.039 (0.755-1.430)
Q3	431/5127	0.886 (0.744-1.056)	94/5127	0.826 (0.569-1.201)	133/5127	0.897 (0.646-1.246)
Q4	510/5133	0.969 (0.817-1.150)	97/5133	0.774 (0.530-1.130)	179/5133	1.167 (0.855-1.594)
Q5	502/5131	0.937 (0.791-1.109)	115/5131	1.117 (0.790-1.580)	156/5131	0.990 (0.720-1.360)
P for trend		0.292		0.972		0.881
**Lunch**						
Q1	317/4541	1	72/4541	1	96/4541	1
Q2	419/4539	1.122 (0.946-1.330)	100/4539	1.122 (0.791-1.592)	119/4539	1.042 (0.761-1.426)
Q3	414/4541	1.077 (0.905-1.280)	80/4541	0.853 (0.587-1.239)	120/4541	1.008 (0.731-1.389)
Q4	413/4540	1.036 (0.869-1.234)	99/4540	1.036 (0.721-1.488)	138/4540	1.112 (0.811-1.525)
Q5	359/4541	0.985 (0.821-1.180)	79/4541	0.959 (0.655-1.402)	114/4541	1.082 (0.781-1.498)
P for trend		0.421		0.724		0.571
**Dinner**						
Q1	421/5567	1	112/5567	1	118/5567	1
Q2	491/5567	0.939 (0.790-1.116)	112/5567	0.848 (0.601-1.197)	140/5567	1.038 (0.745-1.446)
Q3	558/5568	1.068 (0.902-1.264)	121/5568	0.854 (0.604-1.206)	183/5568	1.441 (1.052-1.975)
Q4	521/5567	1.008 (0.850-1.196)	119/5567	0.896 (0.635-1.265)	144/5567	1.066 (0.767-1.483)
Q5	454/5567	0.805 (0.672-0.964)	93/5567	0.591 (0.401-0.870)	151/5567	1.039 (0.744-1.450)
P for trend		0.014		0.013		0.741

Adjustments included age (5 categories), sex, classification of BMI, ethnicity, income, education level, smoking and drinking status, regular exercise, nutrient supplement use, percentage of energy provided by protein, fat and carbohydrates, AHEI (10 categories), the prevalence of diabetes, hypertension, and hyperlipidemia, and medication use for glucose or blood pressure or blood lipids, total intake of energy (10 categories), total intake of dietary fiber (low/high), energy intake at breakfast, lunch and dinner, residual fiber intake at breakfast, lunch and dinner, total vegetable and fruit intake at breakfast, lunch and dinner. BMI, body mass index; AHEI, Alternative Healthy Eating Index; CVD, cardiovascular disease; Case/N, number of case participants/total; Q, Quintile; HR, hazard ratio.
